# Revealing membrane alteration in cells overexpressing CA IX and EGFR by Surface-Enhanced Raman Scattering

**DOI:** 10.1038/s41598-018-37997-3

**Published:** 2019-02-12

**Authors:** Giulia Rusciano, Emanuele Sasso, Angela Capaccio, Nicola Zambrano, Antonio Sasso

**Affiliations:** 1 Department of Physics E. Pancini, University of Naples Federico II, Complesso Univesitario Monte S. Angelo, Via Cintia, I-80126 Naples, Italy; 2 National Institute of Optics (INO)-National Research Council (CNR), Via Campi Flegrei 34, I-80078 Pozzuoli, NA Italy; 3 Department of Molecular Medicine and Medical Biotechnology, University of Naples Federico II, Via S. Pansini 5, I-80131 Naples, Italy; 4 CEINGE Advanced Biotechnologies S.C.aR.L., Via G. Salvatore 486, I-80145 Naples, Italy; 5 Present Address: Nouscom SRL, Rome, Italy

**Keywords:** Membrane biophysics, Applied optics

## Abstract

Sensitive detection of altered proteins expression in plasma membranes is of fundamental importance, for both diagnostic and prognostic purposes. Surface-Enhanced Raman Scattering (SERS) has proven to be a quite sensitive approach to detect proteins, even in very diluted samples. However, proteins detection in complex environment, such as the cellular membrane, is still a challenge. Herein, we demonstrate a SERS-based platform to reveal the overexpression of target proteins in cell membranes. As a proof of concept, we implemented ectopic expression of carbonic anhydrase IX (CA IX) and epidermal growth factor receptor (EGFR) in the plasma membrane of the SKOV3 tumor cell line. Our outcomes demonstrate that SERS signals from cells put in contact with a hyperuniform SERS substrate allow highlighting subtle differences in the biochemical composition of cell membranes, normally hidden in spontaneous Raman confocal microscopy. This opens new opportunities for a label-free membrane analysis and bio-sensing in a broader sense.

## Introduction

The plasma membrane of cells defines the border between the interior of the cell and the outside environment. It rules many critical cell functions, such as cell signalling and trafficking, thanks to the presence of a large variety of membrane proteins (MPs), specifically coordinating selected cell functions. Alteration of this complex biochemical machinery is often associated to serious diseases, including the malignant transformation of cells. It is thus not surprising that MPs are largely studied for therapeutic, diagnostic and drug-delivery related purposes^1,2^. Most of MPs studies involve extraction and solubilisation steps, and are therefore performed outside from the MP native environment^3,4^. Fluorescent tagging is surely a quite sensitive approach for MPs *in situ* detection^5^, but it suffers from some limitations, such as background fluorescence, poor spectral selectivity and photobleaching. Surface enhanced Raman scattering nanoparticles (SERS-NPs), constitute an attractive alternative to fluorescent probes for biological labelling due to their photo-stability and multiplexing capabilities^6–9^. SERS-NPs have been used to reveal the distribution of specific cell surface biomarkers in immunolabelled endothelial cells^10^ and breast cancer cells^11^. However, the use of SERS-NPs is not optimal for live-cell imaging applications, due to NP internalisation and trafficking by cells^11^. Recently, we demonstrated a label-free, SERS based approach for the analysis of erythrocytes membranes^12^, in which unlabelled cells were put in contact with a high uniform and densely packed SERS substrate. Clearly, due to the short-range response of the SERS mechanism^13^, only bio-molecules exposed on the membrane can be efficiently detected, conferring a high-contrast with respect to the bulk Raman contribution^14–16^. It is worth noticing that the spatial uniformity of the plasmonic substrate enhancement factor (EF)^17^ is a crucial parameter of such type of investigation, minimising the intrinsic signal fluctuations in SERS imaging^12^. Herein, we demonstrate a SERS-based approach to reveal the overexpression of a selected protein on the plasma membrane of cells. As proof of concept, we established a model of ectopic expression of two model proteins, carbonic anhydrase IX (CA IX) and epidermal growth factor receptor (EGFR), in the tumour cell line SKOV3. CA IX is a trans-membrane protein, typically expressed in normal cells after hypoxic treatments. Cancer cells constitutively express CA IX, to support the metabolic shift towards anaerobic glycolysis. In particular, CA IX overexpression represents the expedient by which cancer cells manage to neutralise the acidic pH resulting from the anaerobic metabolism. Accordingly, CA IX expression represents a negative prognostic marker in cancer^18^. EGFR is an integral membrane protein, able to elicit intracellular signalling mediated by a tyrosine kinase activity, after binding to cognate ligands in the extracellular environment^19^. Its activity is often de-regulated in cancer, so that EGFR, as well as additional members of its family of tyrosine kinase receptors, are molecular targets for innovative cancer therapeutics^20^.

Our outcomes demonstrate that SERS data from cancer cells placed in contact with a *hyperuniform*^21,22^ SERS substrate, combined with a robust statistical analysis of data, allows to highlight differences in the biochemical composition of cell membranes hidden by spontaneous Raman confocal microscopy. Our results support the use of a SERS-based platform for a label-free membrane analysis, a competence which is of fundamental importance for both diagnostic and prognostic purposes.

## Results and Discussion

### Quantitative Assessment of ectopic expression of CA IX on SKOV3 cell membrane

**Figure 1:**
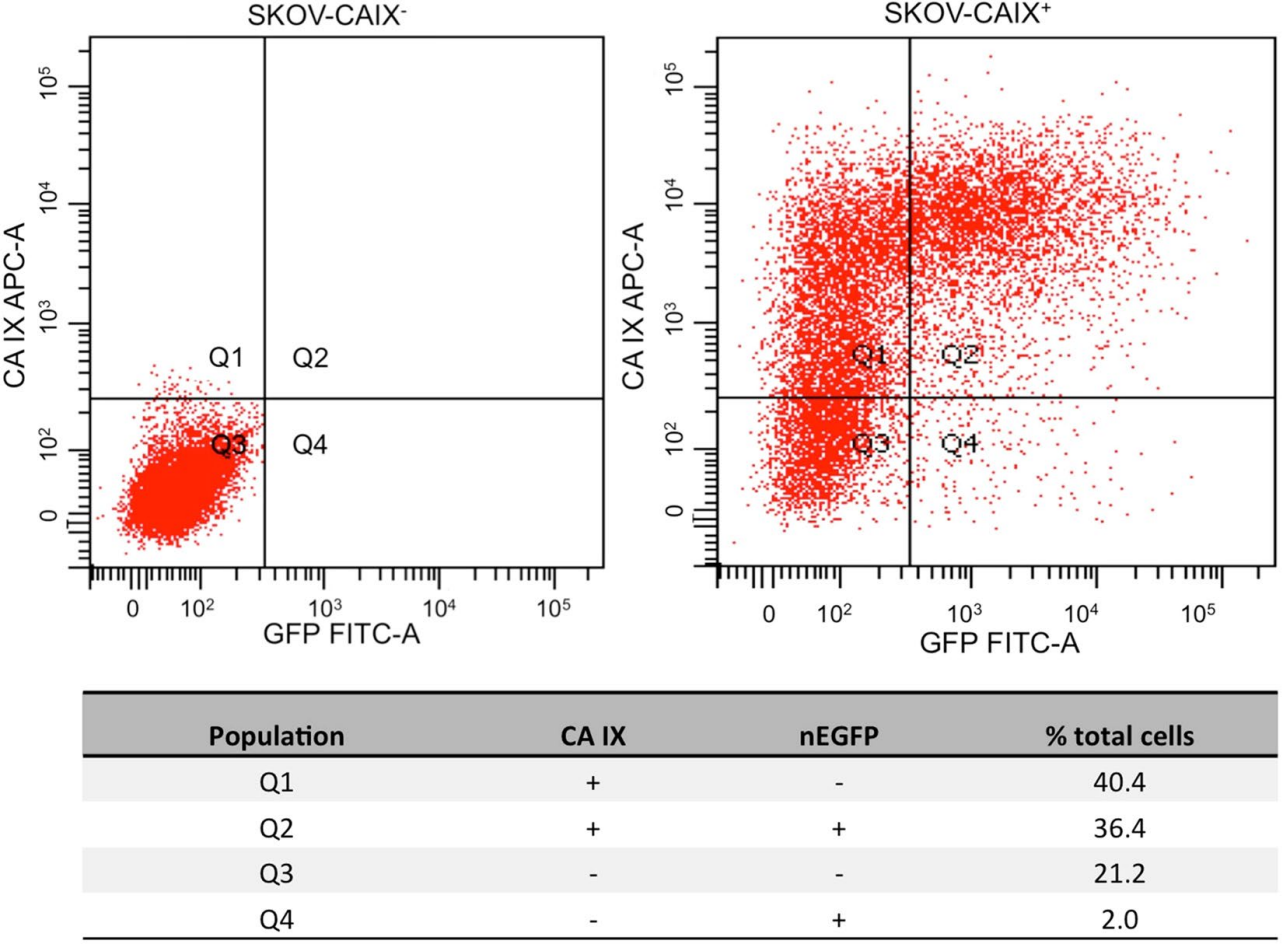
FACS analysis of transfected SKOV3 cell sub-populations. The x axes indicate fluorescence intensity for EGFP expression, while the Y axes show the fluorescence levels of the APC dye (anti-CA IX antibody). The left panel shows untransfected cells for gating purpose. Untransfected SKOV3 cells show very low basal expression of CA IX and absence of auto fluorescence in the nEGFP channel. The Panel on the right shows the FACS pattern of SKOV3 cells transfected with both nEGFP and CA IX expression vectors. The percentage of the different cell populations, gated within the Q1 to Q4 sectors, is reported in the accompanying table. The values of Q2 (36.4%) and Q4 (2.0%) gates show that just 5% of cells within the population of EGFP^+^ cells (Q2 + Q4) can be recognised as false positive for CA IX expression.

In order to demonstrate the suitability of SERS to detect protein expression on cell surface, we selected the tumour cell line SKOV3 for overexpression of the CA IX model protein by transfection. Gene transfection efficiency is affected by several factors^23^. Therefore, in order to face the statistical variability of such procedure and to distinguish successfully transfected cells from untransfected ones, SKOV3 cells were transfected with CA IX in the presence of a vector encoding a fluorescent, EGFP protein (nEGFP), specifically expressed within the nuclear compartment^24^. nEGFP reporter protein was preferred to other versions of the EGFP (*i.e*., cytosolic or membrane-associated), in order to avoid possible interference of a cytoplasmic fluorescence with SERS signal detection at the membrane level. In particular, a 1:10 molar ratio of the expression vectors EGFP:CA IX was selected. Figure 1 shows a typical fluorescence-assisted cell sorting (FACS) profile of co-tranfected cell populations. The counts of cells positive to nEGFP expression (nEGFP^+^), corresponding to gates Q4 and Q2 of Fig. 1, reveals that around 95% of the nEGFP^+^ cells are certainly positive for CA IX expression on the cell surface (in the following indicated as CAIX^+^). Accordingly, visual selection of nEGFP^+^ cells for SERS analysis resulted in a 95% probability of selecting CAIX^+^ cells. In the following, we will indicate as CAIX^−^ SKOV3 cells not transfected with CAIX but successfully transfected with the nEGFP reporter protein. CAIX^−^ cells will be used as *control* sample. It is worth noticing that this choice allows the factorisation of any possible interferences of nEGFP in the acquired signals, as well as any possible cellular stress due to the transfection process.

### SERS-Assay Design and Data Processing

**Figure 2:**
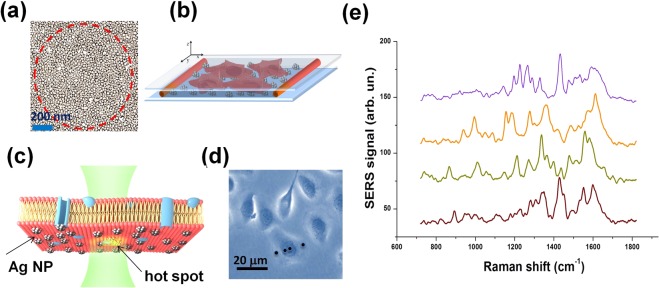
SERS analysis of SKOV3 cells. (**a**) TEM image of a 1 *μ*m × 1 *μ*m Ag nanoislands pattern of our hyperuniform plasmonic substrate. The average nanoislands gap is about 2–3 nm. The dotted line denotes the laser spot area (about 0.8 *μ*m^2^), within which about 400 hot-spot sites occur. This guarantees a high EF value and a very good spatial reproducibility. (**b**) Schematic drawing of the sample cell used for SERS analysis. The structure is comprised of a nano-patterned Ag-substrate covered by a glass coverslip on which SKOV3 cells were cultured. The two layers were glued with low vapour vacuum grease in order to avoid water evaporation. (**c**) Cartoon of SERS data acquisition process. Only molecules at nanometric distance from Ag nanoislands give rise to a detectable SERS signal. (**d**) Bright field image from SKOV3 cells analysed in this work. (**e**) Four background subtrated SERS spectra acquired for the cell shown in (**d**). They were acquired with a 10 *μ*w laser power (on the sample) and an integration time of 2s. Spectra were selected in order to highlight the variability, in terms of bands positions, among the acquired signals owning to the same cell.

SERS measurements on transfected cells were performed with a commercial confocal micro-Raman apparatus (WITec Alpha 300 system), endowed with a Raman probe at 532 nm. This system was modified in order to include an optical branch used to check the fluorescence of SKOV3 cells due to nEGFP expression (see Materials and Methods). *Hyperuniform* plasmonic SERS substrates were prepared according to the procedure fully described in ref.^22^ and shortly summarised in the Materials and Methods paragraph. In Fig. 2a we show a 1 × 1 *μ*m TEM image of our SERS substrate, which approximately corresponds to the area shined by the laser spot. It reveals the dense packing of Ag nanoislands, exhibiting an average interparticles gap of about 2–3 nm. Cells cultured on a glass coverslip were put in contact with the SERS substrate by simply laying down the cell coverslip onto the plasmonic substrate, allowing cells adhesion by gravity. In order to avoid water evaporation, the substrate/coverslip sandwich was sealed-off with a thin layer of low vapour vacuum grease (a schematic representation is given in Fig. 2b). Optimal adhesion of cells on the plasmonic layer, revealed by the presence of high intensity SERS signals, was obtained after a few tens of minutes from sample preparation.

SERS spectra were acquired at different times after transfection: session 1 (48 h), session 2 (60 h), session 3 (72 h) and session 4 (96 h). SERS data acquisition protocol was applied only on CAIX^+^ and CAIX^−^ cells positive to the in-line nEGFP assay. It consisted in the acquisition of SERS signals in N = 20 different points of a line connecting two opposite points of the cell border (see Fig. 2d). Typically, the obtained spectra exhibited a high variability, concerning both intensity and bands spectral positions; an example is shown in Fig. 2e. Given the spatial uniformity of the EF for the employed SERS substrate^12^, there are many causes that can give rise to these fluctuations. The irregular morphology of the cell membrane could be surely an issue, determining changes of the substrate-membrane distance. Moreover, even at a given point, some deleterious impact may be induced by the intermittent contact between membrane and surface at nanometric scale, due to the intrinsic fluidity of the membrane itself^12^. Overall, we deemed it appropriate to carry out spatial and temporal averaging of SERS signals to deal with any unwanted fluctuations. The criteria we followed are described in details in the Material and Methods paragraph.

### Multivariate Analysis of SERS spectra

**Figure 3:**
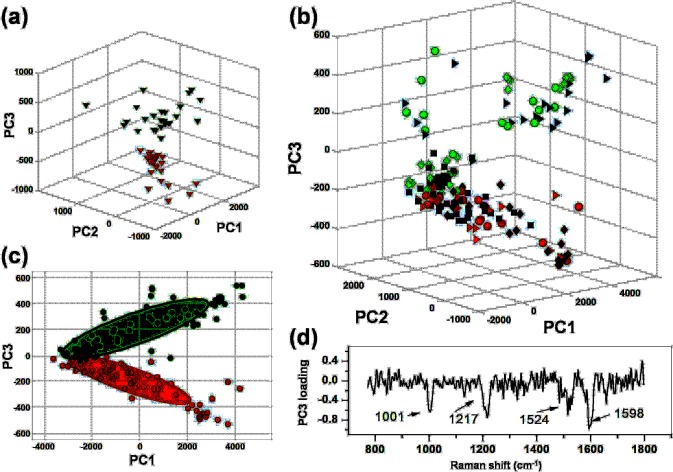
(**a**) PCA score plot obtained in a single measurements session. CAIX^+^ and CAIX^−^ cells are represented by red and green symbols, respectively. (**b**) Score plot obtained from spectra collected in 4 different experimental sessions (dots: session 1; triangles: session 2; diamonds: session 3; squares: session 4). Reddish/greenish colour corresponds to CAIX^+^/CAIX^−^ cells. Differentiation occurs mainly along the PC3 coordinate. (**c**) 2D scatter plot (PC1-PC3 plane) for data shown in part **b** but without distinguishing the four sessions. The 95% confidence ellipses relative to CAIX^+^ (red) and CAIX^−^ (green) are also shown. (**d**) PC3 loading plot from data shown in part **b**. Labels highlight the main spectral feature of PC3 loading.

SERS spectra resulting from the previously mentioned averaging procedure were processed by Principal Component Analysis, PCA^25^. Figure 3a reports the outcomes of PCA obtained for a single measurement session (session 2). As shown, we found a quite good separation between CAIX^+^ cells (red symbols) and CAIX^−^ cells (green symbols). Similar results were obtained for all the other sessions. Intriguingly, a higher clusterization of points was revealed for cells prepared at lower confluence and analysed after a longer time lapse from the transfection process. We speculate that this could be ascribed to a higher presence of daughter cells in the sample, split from parent cells during the mitosis cycles occurred between the two measurement sessions. This reasonably could contribute to homogenise the cellular sample. The capacity to highlight the presence of CA IX on the cellular membrane is still conserved by processing by PCA the spectra collected in all the investigated 4 sessions (see Fig. 3b), although at a slightly lower level with respect to the single session. This is absolutely not an obvious outcome because the complex membrane biochemistry might render intrinsically different samples acquired in different sessions. Figure 3b also shows that a certain clusterization of cells belonging to the same session can still be appreciated, especially along the PC3 score coordinate. Looking at the loading relative to the PC3 component (see Fig. 3d), we notice that all the peaks of this loading can be attributed to protein components, while no lipid-related feature can be observed (*e.g*. in the region around 1440 cm^−1^), suggesting that the difference highlighted by this loading is not amenable to modification of lipids induced by the transfection process.

In order to test the reliability of our SERS based assay of cellular membranes, we used the leave-one-out cross validation (LOOCV) procedure. Calling N the total number of analysed cells, PCA was calculated using N-1 spectra (training set), and the scores of the left-out spectrum was calculated projecting it on the PC vectors of the training data. Therefore, this spectrum was attributed to a given sample by evaluating its position with respect to the 95% confidence ellipse of the training data (whose classification was assured known) in the space of the first three scores. This procedure was iteratively repeated until all the spectra set were left out. Figure 3c reports the 95% ellipse relative to all data. It is worth noticing that these ellipses remain substantially unchanged in the iterative procedure of LOOCV, which suggests a sufficient data sampling of our analysis. The results of LOOCV analysis were summarised by the confusion matrix shown in Table 1. From the elements of this matrix, it was possible to estimate a sensitivity ~94%, a specificity ~93% and a global accuracy ~94% of our SERS-based approach for CAIX detection.

**Table 1:** Confusion matrix giving the classification for CAIX^+^ and CAIX^−^ SKOV3 cells.

True/Predicted	CAIX^+^	CAIX^−^
CAIX^+^	77	5
CAIX^−^	4	76

### Spontaneous Raman *vs* SERS to reveal membrane proteins overexpression

**Figure 4:**
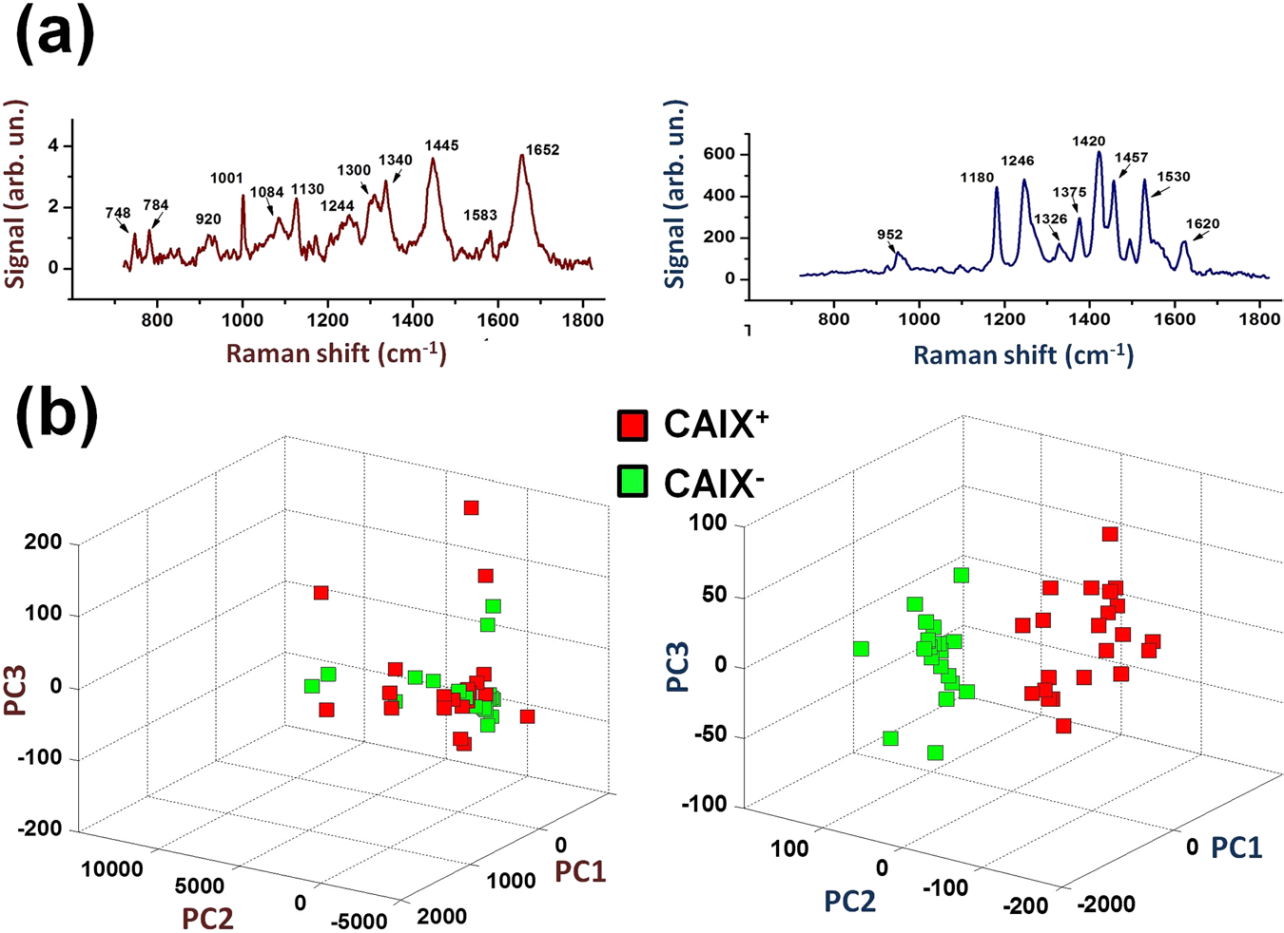
Comparison between Raman and SERS analysis of SKOV3 cells. (**a**) Typical Raman (left) and SERS (right) spectrum. In both cases, the main spectral features are highlighted by labels reporting their spectral position. (**b**) Score plot resulting from the analysis of Raman (left) and SERS (right) spectra from CAIX^+^ (red symbols) and CAIX^−^ (green symbols).

Another interesting question is whether the differences found from SERS could also be observed by the simpler spontaneous Raman spectroscopy (RS). This issue was verified by analysing the same cellular sample, first by RS and then by SERS, by covering the sample with the plasmonic substrate. In both cases, the signal associated to each cell was obtained by averaging 20 signals from a line scan. Figure 4a reports a comparison between a typical Raman (left panel) and SERS (right panel) signal from SKOV3 cells. Notably, both signals were acquired by using the same integration time (2s), while the laser power was three orders of magnitude higher for the spontaneous Raman case. Besides, we remark that Raman signals exhibits DNA related spectral features (such as the peak at 784 cm^−1^) coming from the nuclear region, while all SERS features can be ascribed to lipids and proteins^26^. Figure 4b reports the PCA score plot obtained from both spontaneous Raman (left) and SERS (right) spectra of SKOV3 cells. In both cases, red and green symbols represent CAIX transfected/untrasfected cells, respectively. No difference between CAIX^+^ and CAIX^−^ cells was detected for spontaneous RS measurements for the first three score orders, the same being true for higher orders PC (data not shown). Instead, a complete separation between CAIX^+^ and CAIX^−^ cells was obtained in SERS analysis, highlighting a higher sensitivity of SERS to membrane modifications.

### Insight on the selectivity to proteins overexpression

**Figure 5:**
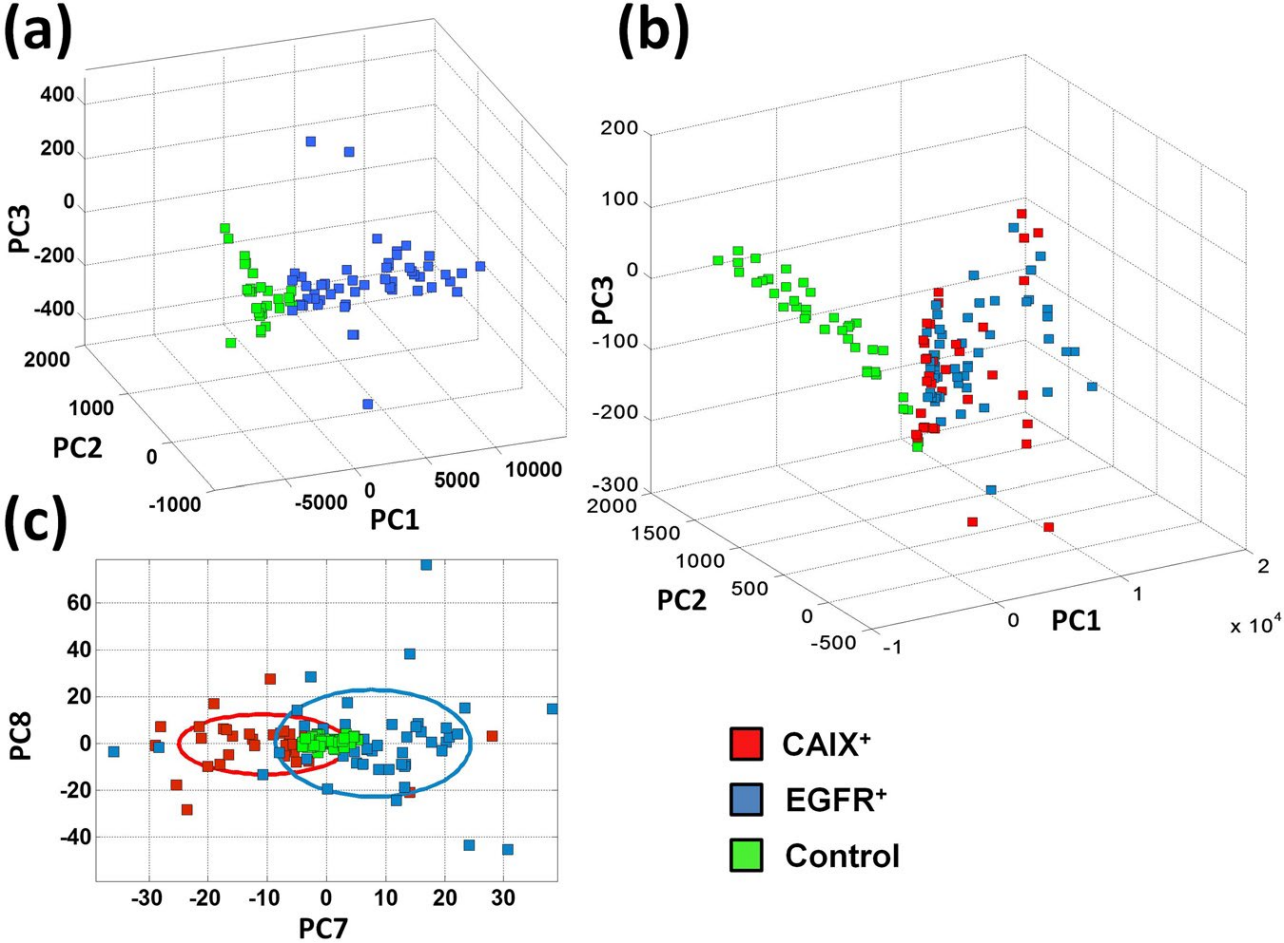
(**a**) PCA score plot obtained by SERS analysis of EGFR^+^ and EGFR^−^ cells, represented by blue and green symbols, respectively. (**b**) Score plot obtained by PC analysis of SERS spectra from EGFR^+^ (blue squares), CAIX^+^ (red squares) and Control (green squares). Globally, the first 3 PCs take into account of ~92% of data variability. (**c**) 2D scatter plot (PC7-PC8 plane) from the same data analysed in (**b**). Samples are differentiated, to a good extent, along the PC7 coordinate, which describes around 2% of variability among samples. The 95% confidence ellipses relative to the three samples are also shown.

To further asses the strength of our SERS based approach to reveal the modification of the chemical pattern of cytoplasmic membrane associated with overexpression of proteins, the same analysis was repeated with another protein, the EGFR. Also for this case, cells were co-transfected with EGFP, in order to solve the statistical uncertainty associated with the transfection process. Again, SERS analysis reveals a quite relevant capacity to distinguish cells overexpressing EGFR (EGFR^+^ cells) with respect to cells transfected in order to express only nEGFP (EGFR^−^), as clearly shown in Fig. 5a. Moreover, as for the CAIX case, we verified that spontaneous Raman is not able to discriminate cells overexpressing EGFP on the cytoplasmic membrane. As a further step, we checked the level of selectivity of our SERS-based approach to distinguish the overexpression of two target proteins. Hence, we applied PCA to CAIX^+^, EGFR^+^ and control cells, which lead to the scatter plot reported in Fig. 5b. Clearly, in this plot, CAIX^+^ and EGFR^+^ cells (red and blue squares, respectively) are separated from the control sample, while no difference was found between CAIX^+^ and EGFR^+^ cells, which, in fact, occupy the same score plot region. As a matter of fact, cells showing CA IX or EGFR expression may result indistinguishable due to elicitation of similar membrane perturbations, which may not be dependent on the size, the protein sequences and the glycosylation patterns of the extracellular domains exposed on the plasma membrane. However, by limiting analysis to only CAIX^+^ and EGFR^+^ cells (therefore not including control cells), a good differentiation between the two different samples is again obtained, with a global accuracy ~90% (data not shown). This outcome suggested us to check the discrimination capability (among CAIX^+^, EGFR^+^ and control) at higher PCs. Discrimination among samples was revealed, to a certain extent, by PC7, a component which describes only ~2% of variation of SERS data. Figure 5c shows this outcome. In the same plot, the 95% confidence ellipses for all the samples are also displayed. It is worth noticing that control cells exhibit a relatively small area in the plot, which suggests a more pronounced homogeneity of control cells with respect to the CAIX^+^ and EGFR^+^ samples. However, it should be noticed that the use of a high order PC (describing therefore only a little variation among data), casts doubts upon the reproducibility of the here obtained experimental outcome.

## Conclusions

Herein, we have demonstrated that SERS analysis of cell membranes is able to reveal membrane alteration induced by overexpression of a model membrane protein. This intrinsically label-free approach could be quite useful in many contests. For instance, it could provide precious information on the dynamic transformation of cells induced by external stresses, including drugs administration. Moreover, SERS could be a quite powerful tool for early diagnosis of overexpression of cancer-related proteins, such as CA IX and EGFR, exposed on the cell membrane. Our SERS-based approach suffer from some limits, also highlighted in this paper, concerning in particular its selectivity. In particular, our approach fails in discriminating among CAIX^+^, EGFR^+^ and control cells, unless pushing PCA to high order, and therefore poor significant, components. Moreover, its efficiency reasonably depends upon the specific protein involved in the investigation. Nevertheless, our results demonstrate the possibility to highlight differences in the protein framework of cellular membranes.

In particular, it is reasonable to envisage a quite good sensitivity of our approach to reveal membrane transformation, since such processes typically involve a consistent change of the membrane protein pattern (*e.g*. malignant transformations). Globally, our results pave the way for a label-free membrane analysis, a fundamental issue for both fundamental biomedical studies and applied researches devoted to sensors development.

## Materials and Methods

### DNA constructs, cell cultures, transfections and FACS analysis

The vector expressing nEGFP, pEGFP NLS-SV40(TAg), was obtained by cloning the T antigen polyA signal from SV40 in the pEGFP C1 vector, as described in ref.^24^; the pRcCMV backbone was used to generate vectors expressing the full length CA IX^27^ and EGFR proteins. SKOV3 cells were cultured in RPMI Medium 1640-GlutaMAXTM-I. The medium was supplemented with 10% heat-inactivated fetal bovine serum (FBS), 50 UI ml^−1^ penicillin, 50 *μ*g ml^−1^ streptomycin, 2mM L-glutamine. All the reagents for cell culturing were from GibcoTM, Thermo Fisher Scientific. Cell lines were purchased from the American Type Culture Collection (ATCC) and cultured in a humidified atmosphere containing 5% CO_2_ at 37.

For SERS analysis, 5 · 10^4^, 7 · 10^4^ or 1 · 10^5^ SKOV-3 cells were seeded in 12 well plate and cultured on sterile round cover glass. 24 hours after seeding, cells were co-transfected with a 1:10 molar ratio of the expression vector pRc/CMV-CA IX encoding CA IX or EGFR (1 *μ*g) and pEGFP-NLS-C1 (100 ng), encoding nEGFP by using 2 *μ*L Lipofectamine Transfection Reagent (Life Technologies, Inc.). As control cell population, SKOV-3 cells were co-transfected with pEGFP-NLS-C1 and empty pRc/CMV vector.

FACS analysis was performed at the CEINGE in-house facility. 1 · 10^6^ SKOV-3 cells were seeded in 100 mm dishes. 24 hours later, cells were co-transfected with 1:10 molar ratio pRc/CMV-CA IX or empty pRc/CMV vectors (10 *μ*g) and pEGFP-NLS-C1 (1 *μ*g) by using 20 *μ*L Lipofectamine. 48 hours after transfection, cells were detached by using PBS EDTA 5 mM, and washed 2 times with PBS. Cells were stained for 30 min with

10 *μ*L of Human Carbonic Anhydrase IX/CA9 APC-conjugated Antibody FAB2188A, R & D System. After 2 washes, APC membrane fluorescence and EGFP were analysed and monitored by cell-sorter Becton Dickinson FACSAria.

### Plasmonic Substrates preparation and characterisation

Plasmonic substrate preparation is described in detail in ref.^22^. Briefly, BCP micelles were prepared starting from 97.2 mg PS-b-P4VP (Polymer Source Inc) dissolved in a solution of 7.0 g of tetrahydrofuran and 10.5 g of toluene taken under stirring at 700 rpm for 3 h at 25, then for 2 h at 67, before slowly cooling down the solution at room temperature. Therefore, micelles were loaded with Ag^+^ by adding 203.7 mg of AgNO_3_ and stirring (700 rpm) for 24 h. Ag^+^ reduction was accomplished by adding drop-wise 440 *μ*l of a solution obtained by dissolving 440 mg of fine dry powder of NaBH_4_ in 4.4 ml of the same solvent THF/toluene used for micelles preparation. After filtering with a 200 nm PTFE syringe filters, this solution was centrifuged at 11 krpm for 20 min to separate the possible unloaded supernatant micelles and sonicated for 22 min. Finally, 100 *μ*l of the resulting solution was left over the supporting glass for 30 s before starting the spin-coating at 1.0 krpm speed for 60 s. This produced an uniform film coating of nanoislands with an average size of ~26 nm and an interparticles gap ~2–3 nm. Finally, UV exposure for ~20 h at 254 nm (Hg lamp, Sankyo Denki G15T8) at an energy density of 1.7 mJ cm^−2^, removed the copolymer. The achieved reddish plasmonic film on glass appeared as a transparent optical coating with a green backscattering glow.

In order to estimate the EF factor, we used Crystal Violet (CV) as probe molecule. Therefore, we measured both spontaneous Raman and SERS signals from a proper CV aqueous solution. In order to detect SERS signals produced by a controlled number of molecules, we set-up an evaporation cell constituted by two parallel substrates (24 × 24 mm) distanced by 4.5 *μ*m beads used as spacers, and sealed. Therefore, a volume of 100 *μ*l of a 380 nM CV solution was infiltrated in this cell and allowed to dry slowly. In this way, a nearly uniform CV film is produced, in which N_*surf*_ = 218 molecules are present within the scattering area of 0.44 *μ*m^2^. SERS signal *I*_*SERS*_ was acquired by using an impinging power (on the sample) of *P*_*SERS*_ = 4 *μ*W and an integration time of 2s. Then, we acquired the spontaneous Raman signal (*I*_*Raman*_) from a 5.57 mM CV aqueous solution, by using a power *P*_*Raman*_ = 0.5 mW and an integration time of 2s. This signal results from N_*vol*_ = 7 × 10^6^ molecules present in the confocal detection volume (~2 *μ*m^2^). The EF was therefore estimated as:

1 $$EF=\frac{{I}_{SERS}}{{I}_{Raman}}\times \frac{{N}_{vol}}{{N}_{surf}}\times \frac{{P}_{Raman}}{{P}_{SERS}}$$

which provided us the value EF = 1.2 × 10^7^. To test the EF spatial reproducibility test, SERS signal was acquired over 50 mm × 50 times region, with a 500 nm step. These measurements allowed to estimate a spatial uniformity of ~5% on this scale.

### Confocal Microscopy

**Figure 6:**
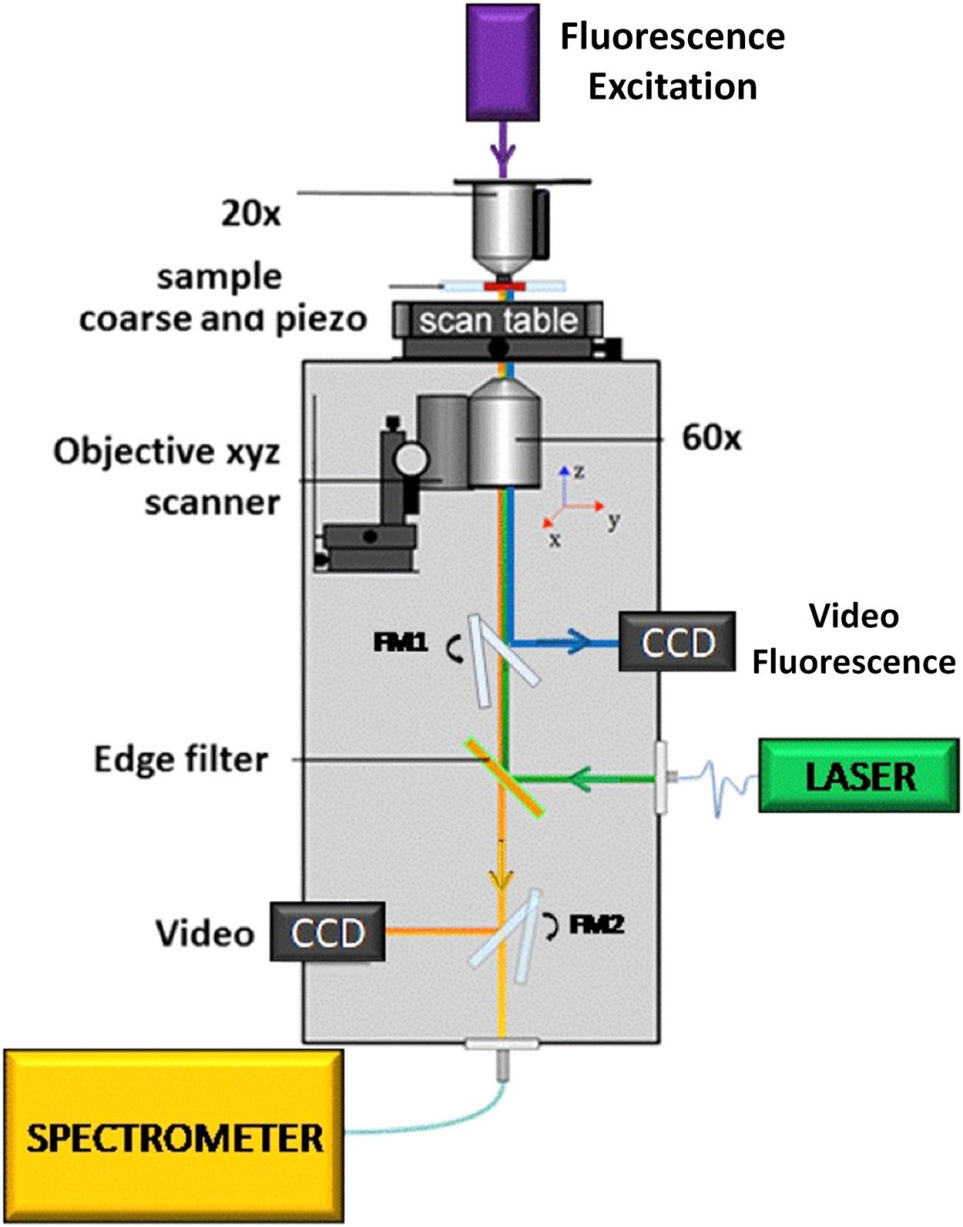
Schematic representation of the Raman/fluorescence optical set-up used for this investigation.

Raman spectra were acquired by using a commercial Raman system (WITec, alpha 300). It consists in an inverted confocal microscope endowed with a Raman excitation source at 532 nm. This laser is focused on the sample through a 60X dry objective (NA = 0.8), providing an almost diffraction limited spot on the sample. In-elastically back-scattered radiation was guided toward the spectrograph by a 50 *μ*m core optical fibre, which assures the system confocality. In order to verify fluorescence of cells induced by GFP transfection, an optical branch devoted to fluorescence excitation/detection was added, as depicted in Fig. 6. In brief, blue light (470 nm) from a fluorescence optical fibre (M470L3-C1) was firstly properly collimated and spectrally filtered, than it was injected into the same microscope objective used as condenser for bright field illumination. Cell fluorescence imaging was accomplished by using the same inverted microscope objective used for Raman excitation, coupled to a CCD camera (Hamamatsu C5985) through a switchable mirror. Samples consisted in culture coverslips, covered by cells at a confluence of ~50% and put in contact with the plasmonic substrate. In order to avoid water evaporation, this sandwich was glued by vacuum grease. Measurements were performed by acquiring 20 spectra on a line connecting two opposite cell borders. Each spectrum was acquired by using an integration time of 2s and an impinging power (on sample) of 10 *μ*W. Spectra were acquired over the spectral range from 700–1800 cm^−1^ (1024 points), with a spectral resolution of ~1.5 cm^−1^. In order to better highlight SERS bands, acquired signals were background-subtracted. Background mainly originates from the presence of polymeric residues on the Ag-nanostructures not completely eliminated by substrate exposure to UV lamp. It presents two quite broad bands in the 1300–1600 cm^−1^ region, which can be easily eliminated by a polynomial fit.

### Data processing

**Figure 7:**
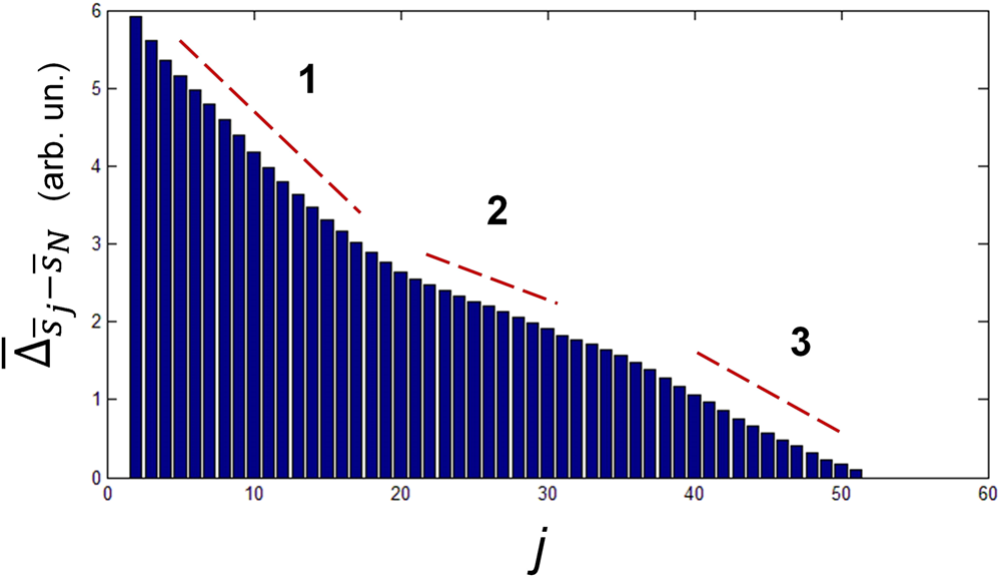
Typical behaviour of ${\bar{{\rm{\Delta }}}}_{{\bar{s}}_{j}-{\bar{s}}_{N}}$ as function of *j*.

The ability to distinguish by SERS the effect of overexpression of a single protein at membrane level is based on the assumption that it is possible to assign to each cell a single SERS spectrum, holding information on the complex molecular structures of its membrane. This is absolutely not an easy task. As a matter of facts, several co-factors should be taken into account. A first source of spectra variability is due to the intermittent contact of the fluid cell membrane with the SERS substrate. This kind of fluctuations can be reduced by a proper choice of the integration time (2s in our case) which provides a temporal averaging. A second important source of fluctuations arises from the membrane heterogeneity which usually occurs at length scale below 200 nm (due to the presence, *e.g*., of lipid rafts). Part of these fluctuations are attenuated by the averaging effect produced by the diffracted laser spot area (1 *μ*m^2^). This average changes, however, moving from point to point on the cell membrane. For this reason, we performed a preliminary study to face this issue. We proceeded by defining a distance ${{\rm{\Delta }}}_{{s}_{a},{s}_{b}}$ between two spectra *s*_*a*_ and *s*_*b*_ acquired in two different points of the cell membrane, according to the relation:

2 $${{\rm{\Delta }}}_{{s}_{a},{s}_{b}}=\frac{1}{{N}_{p}}\sqrt{{\sum }_{i=1}^{{N}_{p}}{({s}_{a,i}-{s}_{b,i})}^{2}}$$

the index *i* running on all the *N*_*p*_ spectral points (wavenumbers). Therefore, we acquired, for a single cell, a line scan of *N* = 50 spectra and repeated the same procedure over 30 cells. *N* = 50 was chosen as a compromise to minimise cellular stress due to the laser exposure. Each spectrum was normalised to the most prominent peak and cleaned up by spurious effects (cosmic rays). Hence, we evaluated the distance ${{\rm{\Delta }}}_{{\bar{s}}_{j}-{\bar{s}}_{N}}$, *i.e*. the difference between the average from all the 50 spectra of the scan (${\bar{s}}_{N}$) and the average spectrum calculated over a set of *j* spectra (*j* < *N*), extracted from all the possible $$(\begin{array}{c}N\\ j\end{array})$$ combinations. Finally, for *j* ≥ 2, we evaluated ${\bar{{\rm{\Delta }}}}_{{\bar{s}}_{j}-{\bar{s}}_{N}}$, value obtained by averaging ${{\rm{\Delta }}}_{{\bar{s}}_{j}-{\bar{s}}_{N}}$ over all the possible combinations. Figure 7 shows the typical behaviour of ${\bar{{\rm{\Delta }}}}_{{\bar{s}}_{j}-{\bar{s}}_{N}}$ as function of *j*. Notably, it is possible to distinguish three regions, characterised by different slopes. The first one, for 2 < *j* < 16, exhibiting the highest slope, reflects a strong variability among spectra, so that ${\bar{s}}_{j}$ differs significantly (in terms of relative intensity of Raman features) from ${\bar{s}}_{N}$, so that it cannot be considered representative of the whole cell. For 16 < *j* < 36, the slope decreases because of the assessment of the relative intensity of the different spectral features. Finally, in the third region, j ≥ 36, approaches to zero (according to its definition) in a fashion depending mainly by the noise superimposed on the spectra. A similar behaviour was obtained for all the 30 analysed cells. In particular, we verified that, for all the 30 cells, ${\bar{{\rm{\Delta }}}}_{{\bar{s}}_{j}-{\bar{s}}_{N}}$ was always in the second region for *j* = 20, meaning that a good representation of the single cell is provided by averaging 20 SERS spectra of the scan. This outcome provided us the criterium to handle the SERS fluctuations in all the measurements shown in this work.

### Handling of interference from culture medium

**Figure 8:**
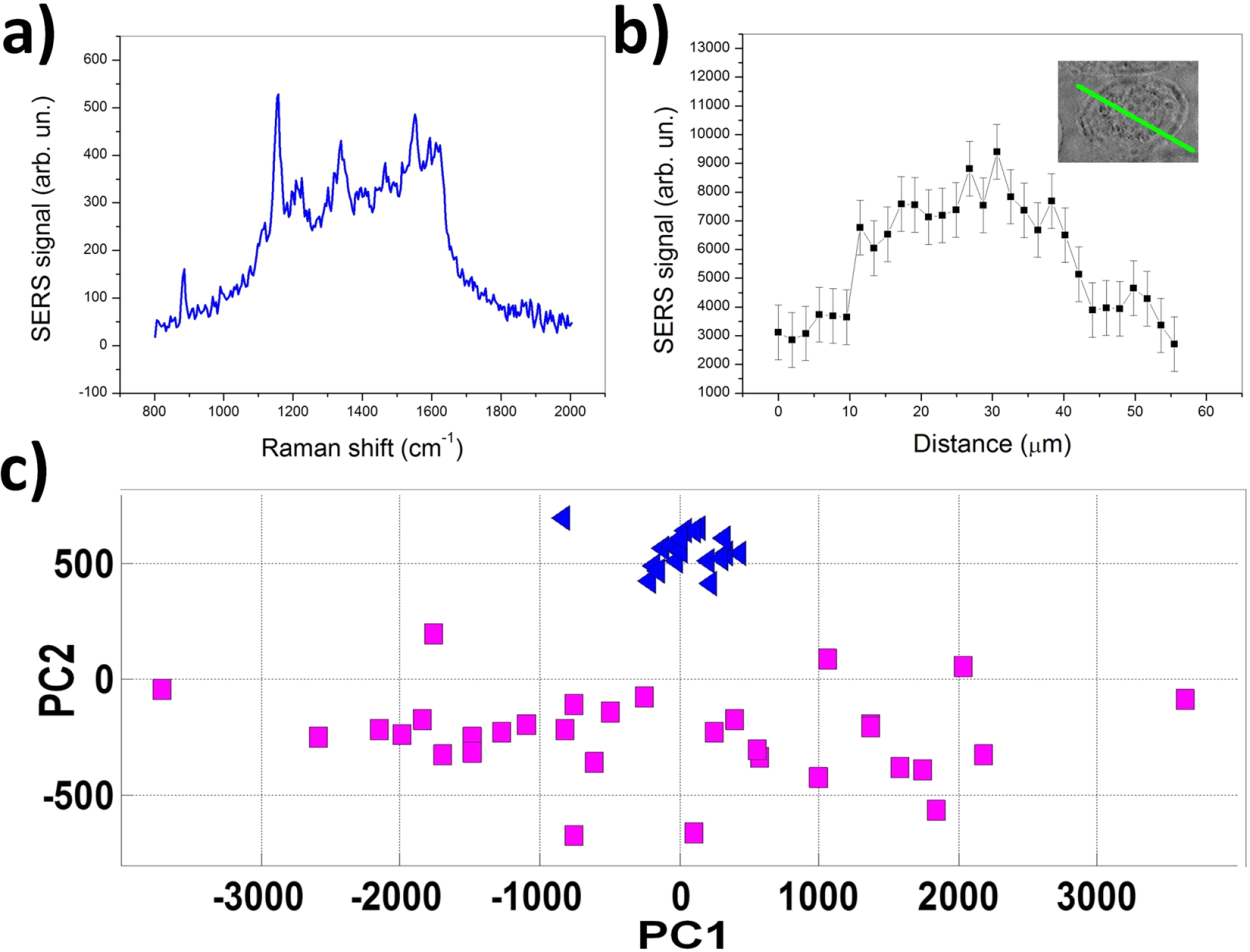
(**a**) A typical SERS spectrum acquired in the medium. (**b**) Integrated intensity of SERS signal acquired along a line across a cell, as shown in the inset. (**c**) PCA performed on SERS spectra acquired in the cell medium (blue triangles) and CAIX^−^ cells (purple squares). Notably, spectra from the medium, which exhibit a relatively high similarity, occupy a quite reduced space in the PC1-PC2 score plot.

An important issue to be considered for SERS spectra analysis concerns the role of the protein-rich medium (PBS, eventually mixed with cells secretions) in which cells lie during measurements. This medium gives rise to a detectable SERS signal (see Fig. 8a), clearly identified in this analysis. Hence, by acquiring SERS signals along a line through the cell, starting and terminating in the medium, it is possible to obtain a SERS profile as shown in Fig. 8b. This SERS profile corresponds to the integrated intensity of Amide I band (1620–1680 cm^−1^). Obviously, the interference from this signal becomes important in the case of poor contact of the cell with the substrate. To face this issue, cells were allowed to adhere to SERS substrate for ~30 min before analysis. Moreover, signals were acquired in the thicker cell region (nuclear region) were cells adhesion was best achieved. In these conditions, a quite good differentiation between signal form cells and from medium was achieved (see Fig. 8).

## Supplementary information

Supplementary Material
