# Publisher Correction: Revealing membrane alteration in cells overexpressing CA IX and EGFR by Surface-Enhanced Raman Scattering

**DOI:** 10.1038/s41598-019-45208-w

**Published:** 2019-06-18

**Authors:** Giulia Rusciano, Emanuele Sasso, Angela Capaccio, Nicola Zambrano, Antonio Sasso

**Affiliations:** 1 Department of Physics E. Pancini, University of Naples Federico II, Complesso Univesitario Monte S. Angelo, Via Cintia, I-80126 Naples, Italy; 2 National Institute of Optics (INO)-National Research Council (CNR), Via Campi Flegrei 34, I-80078 Pozzuoli, NA Italy; 3 Department of Molecular Medicine and Medical Biotechnology, University of Naples Federico II, Via S. Pansini 5, I-80131 Naples, Italy; 4 CEINGE Advanced Biotechnologies S.C.aR.L., Via G. Salvatore 486, I-80145 Naples, Italy; 5 Present Address: Nouscom SRL, Rome, Italy

Correction to: *Scientific Reports* 10.1038/s41598-018-37997-3, published online 12 February 2019

The original version of this Article contained an error in the title of the paper, where the phrase “cells overexpressing” was incorrectly given as “cellsoverexpressing”. This has now been corrected in the PDF and HTML versions of the Article.

