# Supplementary material for: Revealing membrane alteration in cells overexpressing CA IX and EGFR by Surface-Enhanced Raman Scattering
Source: Sci Rep. 2019 Feb 12;9:1832. doi: 10.1038/s41598-018-37997-3 (PMC6372785; doi:10.1038/s41598-018-37997-3)
Supplement: Supplementary file 1 — Supplementary Material [file 41598_2018_37997_MOESM1_ESM.pdf]

Revealing membrane alteration in cells overexpressing CA IX and EGFR by Surface-Enhanced Raman Scattering

Giulia Rusciano[1,2,*,+], Emanuele Sasso[3,4,+], Angela Capaccio[1], Nicola Zambrano[3,5,*], and Antonio Sasso[1,2]

Supplementary Material

Multivariate analysis

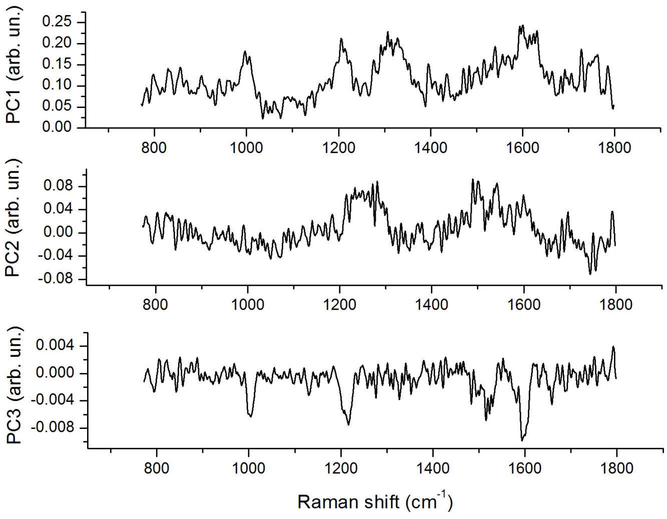

Fig S1: Loading plot for the three first PC components resulting from principal component analysis of CAIX$^+$/CAIX$^-$ spectra.

In Fig. S1 we report the loading plot resulting from PCA of CAIX+/CAIX- spectra. As clearly visible in Fig. 3 of the manuscript, cells are differentiated for the expression of CAIX on membrane only along PC3. Generally specking, it is not possible to choose in advance the correct PC to be used for sample differentiation. This is because, in principle, cells can be differentiated for many reasons (not only the one desired by the researcher). For instance, cells could be different for some form of uncontrolled stress they are exposed to (for instance, heating, mechanical handling, etc). In this case, assuming that this stress causes a membrane modification detectable by SERS, it is clear that PCA will highlights this difference and, as a consequence, stressed cells are separated by the not-stressed ones along a coordinate of the score plot. Clearly, more heterogeneous the sample is, more difficult is to highlight the desired difference (CAIX overexpression in our case).

SERS spectra

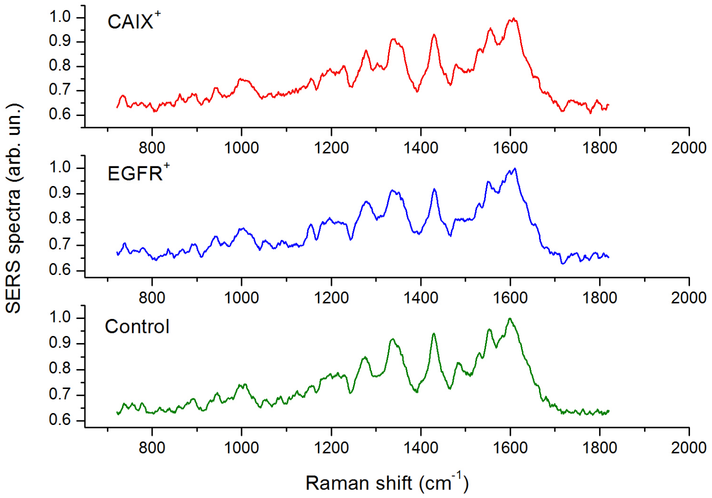

Fig S2: Typical SERS spectra for CAIX$^+$, EGFR$^+$ and control cells.

In Fig. S2 we report typical SERS spectra for CAIX$^+$, EGFR$^+$ and control cells. It is worth noticing that the fluctuation of the relative intensities of bands among samples is within the variability among cells of the same sample. Samples can be differentiated each other only by multivariate analysis (PCA).
